# Atypical Presentation of Stenotrophomonas maltophilia Stomatitis Post COVID-19 in an Immunocompetent Adolescent

**DOI:** 10.7759/cureus.68464

**Published:** 2024-09-02

**Authors:** Mohamed Alharami, Ahmad Anbar, Mohamed Alsharif, Frhaan Zahrawi, Ammar Hemaidan

**Affiliations:** 1 Internal Medicine, Alexandria University School of Medicine, Alexandria, EGY; 2 Gastroenterology, Advanced Medical Research Center, Port Orange, USA; 3 Internal Medicine, University of Aleppo School of Medicine, Aleppo, SYR; 4 Neurosurgery, Alfaisal University College of Medicine, Riyadh, SAU; 5 Gastroenterology, Florida State University College of Medicine, Daytona Beach, USA

**Keywords:** covid-19-related immune dysregulation, child and adolescent health, opportunistic bacterial infection, immuno-competent, oral ulcer, covid-19, stenotrophomonas maltophilia infection

## Abstract

*Stenotrophomonas maltophilia* (S. *maltophilia*) is a rare, multi-drug-resistant opportunistic bacteria that typically causes serious infections in immunocompromised and hospitalized patients, often leading to fatal pneumonia or bacteremia. We present the case of a healthy 15-year-old immunocompetent female who developed severe oral ulcers due to *S. maltophilia* following a recent COVID-19 infection. To our knowledge, this is the first reported case of *S. maltophilia *manifesting in this manner after COVID-19. Scrapes from the oral lesions were collected and cultured, confirming the* *infection.The CBC and immunoglobulin reports revealed mildly elevated IgA and platelet levels, with no evidence of immunodeficiency.The patient was treated with trimethoprim/sulfamethoxazole (TMP-SMX) based on culture sensitivity, and she responded well to treatment. She was referred to an infectious disease specialist for further monitoring. COVID-19 has recently been implicated in many unusual presentations, associations, and syndromes. One of the most supported theories about COVID-19 is its association with a transient immunodeficient state or a generalized state of immune system dysregulation that can compromise both innate and adaptive immunity. This case supports that theory, as no other apparent etiology for such an otherwise opportunistic infection was identified. The recognition of such atypical infections in previously healthy individuals, particularly post COVID-19, highlights the importance of sharpening clinical vigilance and considering opportunistic pathogens in differential diagnoses. Further research is warranted to explore the potential link between COVID-19 and the susceptibility to rare opportunistic infections, which may guide future clinical practices.

## Introduction

*Stenotrophomonas maltophilia* (*S. maltophilia*), a Gram-negative bacillus, is recognized as a multidrug-resistant opportunistic pathogen that has recently been associated with high morbidity and mortality [1-4]. The SARS-CoV-2 virus, responsible for COVID-19, has emerged as a major pathogen causing severe pneumonia over the past few years. *Stenotrophomonas maltophilia* is particularly known for causing serious respiratory tract infections, with pneumonia-associated bacteremia being a leading cause of death in infected patients. Concomitant infections with both *S. maltophilia* and COVID-19 have been reported, often resulting in a severe clinical course characterized by rapidly worsening symptoms, progression to respiratory failure, shock, and multi-organ failure [5-8]. Patients with underlying pulmonary conditions, such as chronic obstructive pulmonary disease (COPD) or those with central venous access, are typically at higher risk for this co-infection, which can lead to exacerbations requiring mechanical ventilation or bacteremia resulting in septic shock. However, what makes this case unique is not only the unprecedented presentation of *S. maltophilia* but also the demographic and immunocompetent status of the patient. Unlike previously documented cases involving *S. maltophili*a in the context of severe pulmonary conditions, our case involves a healthy 15-year-old immunocompetent female who developed severe oral ulcers following a COVID-19 infection. This atypical manifestation in an otherwise healthy individual underscores the potential for *S. maltophilia* to cause significant morbidity outside the traditional high-risk groups. It suggests that COVID-19 may induce a transient state of immune dysregulation, predisposing even healthy individuals to opportunistic infections. Reporting this case contributes to the evolving understanding of post-COVID-19 complications and underscores the need for heightened awareness of atypical infections during the post-viral period.

## Case presentation

**Figure 1:**
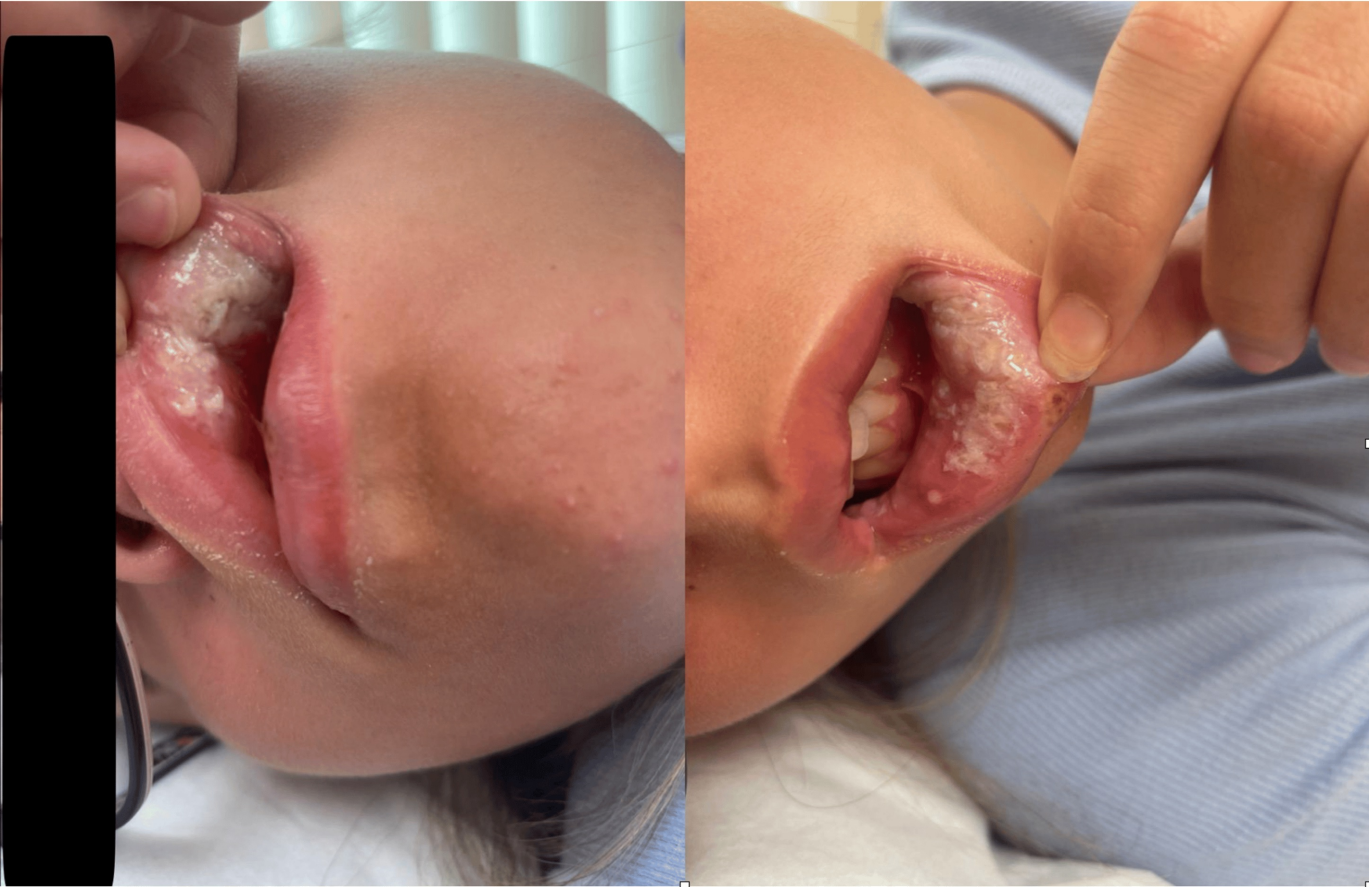
Severe oral lesions on the patient's upper and lower lips were noted on the initial presentation

A 15-year-old female presented to the urgent care unit with her mother complaining of multiple painful oral lesions. Examination revealed multiple oral ulcers of various sizes involving the upper lip, lower lip, and hard palate (Figure 1).

The patient appeared in marked distress and reported pain exacerbated by chewing and swallowing. On physical examination, her weight was 52 kg (BMI 21.6 kg/m^2^), blood pressure was 125/75 mmHg, heart rate was 78 beats per minute, temperature was 98.6°F (37°C), respiratory rate was 16 breaths per minute, and oxygen saturation was 98% on room air. Immunizations were up to date, including the COVID-19 vaccination with a booster. The mother noted that the lesions had developed over the past week, increasing in number and severity. She also mentioned a history of COVID-19 infection about two weeks prior, during which both the patient and her mother experienced mild flu-like symptoms and tested positive for COVID-19 via a swab test. The patient lives with her mother, and no sick contacts were reported. A repeat COVID-19 test was performed, yielding a negative result. The medical history was otherwise insignificant. Multiple scrapes from the lesions were collected and sent for culture. The patient was initially prescribed a one-week prophylactic course of cephalexin while awaiting culture results. One week later, the patient returned with worsening symptoms, and the culture result was positive for *S. maltophilia*, which was sensitive to trimethoprim/sulfamethoxazole (TMP-SMX) and levofloxacin but resistant to all other tested antibiotics. Consequently, the patient was switched to a 10-day course of TMP-SMX. CBC and quantitative immunoglobulin tests were ordered to rule out possible immunodeficiency; all results were negative except for mildly elevated IgA and platelet levels (Table 1).

**Table 1:** Complete blood count and quantitative immunoglobulin levels of the patient Values in bold indicate mildly elevated IgA and platelet levels.

Analyte	Value	Reference range
Immunoglobulin A (IgA)	266 mg/dL	36-220 mg/dL
Immunoglobulin G (IgG)	1069 mg/dL	500-1590 mg/dL
Immunoglobulin M (IgM)	153 mg/dL	41-170 mg/dL
White blood cells (WBCs)	7.7 Thousand/uL	4.5-13.0 Thousand/uL
Red blood cells (RBCs)	4.50 Million/uL	3.80-5.10 Million/uL
Hemoglobin (Hb)	12.6 g/dL	11.5-15.3 g/dL
Hematocrit (Hct)	37.8%	34.0-46.0 %
Mean corpuscular volume (MCV)	84.0 fL	78.0-98.0 fL
Mean corpuscular hemoglobin (MCH)	28.0 pg	25.0-35.0 pg
Mean corpuscular hemoglobin concentration (MCHC)	33.3 g/dL	31.0-36.0 g/dL
Red cell distribution width (RDW)	12.9%	11.0-15.0 %
Platelet count	408 Thousand/uL	140-400 Thousand/uL
Mean platelet volume (MPV)	10.1 fL	7.5-12.5 fL
Absolute neutrophils	3727 cells/uL	1800-8000 cells/uL
Absolute lymphocytes	2926 cells/uL	200-900 cells/uL
Absolute monocytes	585 cells/uL	200-900 cells/uL
Absolute eosinophils	362 cells/uL	15-500 cells/uL
Absolute basophils	100 cells/uL	0-200 cells/uL
Neutrophils	48.4%	40-60%
Lymphocytes	38%	20-40%
Monocytes	7.6%	2-8%
Eosinophils	4.7%	1-6%
Basophils	1.3%	0-2%

**Figure 2:**
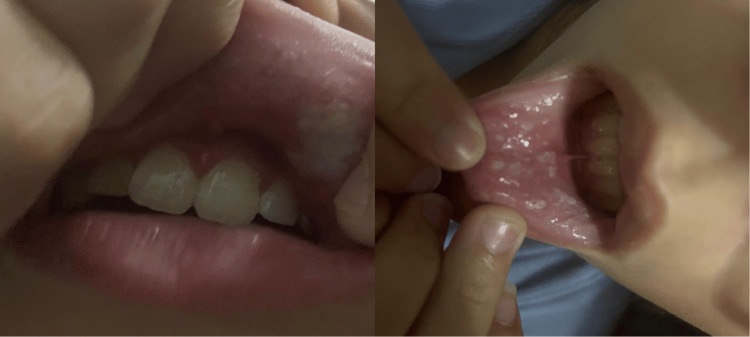
Marked improvement observed after completing the TMP-SMX course TMP-SMX: trimethoprim/sulfamethoxazole

Two weeks later, the patient returned for follow-up and showed dramatic improvement. However, a few lesions were still appreciated and appeared to be healing (Figure 2).

Multiple scrapes were retaken and cultured. The second culture did not show *S. maltophilia* growth but revealed the growth of *Prevotella* species, a normal oral flora. The patient was referred to an infectious disease specialist for further follow-up.

## Discussion

According to a large retrospective cohort study investigating the prevalence of antibiotic resistance in *S. maltophilia* [9], TMP-SMX remains the drug of choice. However, reports of resistance are increasing, estimated at around 12.1%. Levofloxacin is another possible treatment, with an even lower resistance rate, estimated at approximately 8.99%. In this study, 16.0% of bacteremia isolates were resistant to TMP-SMX, and among these, 32.7% were also resistant to levofloxacin, amikacin, tigecycline, and colistin. Consequently, 5.87% of all isolates were classified as extensively drug-resistant (XDR).

Another interesting cohort study [10], found that *S. maltophilia *could be present in the oropharyngeal microbiomes of healthy individuals. The study identified a unique risk for *S. maltophilia* infection in leukemia patients receiving chemotherapy with a history of extensive antibiotic use [10]. These patients may harbor the bacteria as part of their normal oropharyngeal flora and should be monitored for potential *S. maltophilia* infection, particularly following a leukemia diagnosis and during chemotherapy, broad-spectrum antibiotic therapy, or both. It is believed that cumulative antibiotic use, especially of carbapenems, can trigger an infection by *S. maltophilia* in patients with hematological malignancies like acute myeloid leukemia [10]. This correlation suggests that real-time monitoring of the oral cavity may help identify leukemia patients at risk of *S. maltophilia* infection.

Furthermore, another cohort study [11] found that Gram-negative bloodstream infections (GN-BSIs) were reported in 337 patients with hematological malignancies or hematopoietic stem cell transplants (HSCT). Among these, 21 patients had *S. maltophilia* bacteremia, with five (23.8%) also suffering from mucositis. In contrast, among the remaining 316 patients with GN-BSIs other than *S. maltophilia*, only 24 had mucositis (7.6%).

Finally, based on two different cohort studies [12,13], *S. maltophilia* bacteremia in pediatric patients carries a significantly concerning high mortality rate, approaching 34%. Children at high risk are those with prior ICU admission, neutropenia, or a history of broad-spectrum antibiotic exposure. These studies concluded that early identification of children at risk, as well as early diagnosis and prompt treatment of *S. maltophilia* bacteremia, is crucial for survival in this age group.

Based on existing literature [9-13], localized infections such as *S. maltophilia* stomatitis in pediatric patients, particularly those at high risk, warrant thorough investigation to enable early detection and treatment before the infection becomes systemic and potentially fatal. Additionally, clinicians should be vigilant when encountering pediatric patients with prior ICU admissions, neutropenia, or extensive antibiotic exposure, as they are at heightened risk of *S. maltophilia* bacteremia. For patients with newly diagnosed leukemia, screening for* S. maltophilia* in the oral flora before initiating chemotherapy or broad-spectrum antibiotics may be prudent. In those with hematological malignancies or undergoing HSCT, any form of mucositis should be promptly evaluated to rule out a possible *S. maltophilia* bloodstream infection, given its associated high mortality rate. To summarize, *S. maltophilia* is classically associated with immunocompromised adults and hospitalized patients, making its occurrence in a healthy 15-year-old immunocompetent female particularly unexpected. The unusual presentation of painful oral ulcers following a recent COVID-19 infection adds further peculiarity to this case.

One possible explanation for this infection could be undetected immunological abnormalities in the patient. While standard immunological tests, such as CBC and immunoglobulin levels, were within normal limits except for mildly elevated IgA and platelets, it is possible that more subtle or transient immune dysregulation, potentially triggered by the recent COVID-19 infection, contributed to her susceptibility [14,15]. The immune dysregulation that affects the adaptive immune system specifically could play a role in this case, where COVID-19 has been observed to be associated with profound lymphopenia, particularly affecting CD4+ and CD8+ T cells [14]. This depletion not only hinders the body’s ability to clear the virus but also increases the risk of secondary bacterial infections, which this patient might have experienced. Future investigations could explore whether transient post-viral immune suppression or dysregulation plays a role in facilitating opportunistic infections like *S. maltophilia* in otherwise healthy individuals [14,15]. Environmental factors should also be considered. The patient’s environment, including possible exposure to hospital settings or prior antibiotic use, could have contributed to the infection. Further studies might focus on the role of environmental reservoirs of *S. maltophilia* in community settings, particularly following a possible trigger such as viral illnesses like COVID-19.

## Conclusions

*Stenotrophomonas maltophilia* infection in an otherwise healthy adolescent warrants further investigation to identify any possible underlying causes. In this case, a recent COVID-19 infection may have triggered an immune response that became dysregulated, resulting in a temporary immunodeficient state and increased susceptibility to infections. Another possibility is that the SARS-CoV-2 virus itself caused damage to the oral mucosal epithelium, leading to mucosal barrier dysfunction and reduced local innate immunity. However, further research is necessary to establish a definitive relationship between COVID-19 and *S. maltophilia*. Clinicians should maintain a high index of suspicion for atypical presentations of opportunistic infections, even in immunocompetent patients, particularly in the post-COVID-19 period. In cases like this, early screening and monitoring for *S. maltophilia* should be considered in patients presenting with unusual symptoms, especially if they have a recent history of viral infection. Moreover, awareness of this potential complication could prompt stricter follow-up and possibly even prophylactic measures in patients recovering from COVID-19.
